# The Development and Application of a Multiple Gene Co-Silencing System Using Endogenous *URA3* as a Reporter Gene in *Ganoderma lucidum*

**DOI:** 10.1371/journal.pone.0043737

**Published:** 2012-08-24

**Authors:** Dashuai Mu, Liang Shi, Ang Ren, Mengjiao Li, Fengli Wu, Ailiang Jiang, Mingwen Zhao

**Affiliations:** College of Life Sciences, Nanjing Agricultural University, Key Laboratory of Microbiological Engineering of Agricultural Environment, Ministry of Agriculture, Nanjing, Jiangsu, People's Republic of China; University Health Sciences Center, United States of America

## Abstract

*Ganoderma lucidum* is one of the most important medicinal mushrooms; however, molecular genetics research on this species has been limited due to a lack of reliable reverse genetic tools. In this study, the endogenous orotidine 5′-monophosphate decarboxylase gene (*URA3*) was cloned as a silencing reporter, and four gene-silencing methods using hairpin, sense, antisense, and dual promoter constructs, were introduced into *G. lucidum* through a simple electroporation procedure. A comparison and evaluation of silencing efficiency demonstrated that all of the four methods differentially suppressed the expression of *URA3*. Our data unequivocally indicate that the dual promoter silencing vector yields the highest rate of *URA3* silencing compared with other vectors (up to 81.9%). To highlight the advantages of the dual promoter system, we constructed a co-silencing system based on the dual promoter method and succeeded in co-silencing *URA3* and *laccase* in *G. lucidum*. The reduction of the mRNA levels of the two genes were correlated. Thus, the screening efficiency for RNAi knockdown of multiple genes may be improved by the co-silencing of an endogenous reporter gene. The molecular tools developed in this study should facilitate the isolation of genes and the characterization of the functions of multiple genes in this pharmaceutically important species, and these tools should be highly useful for the study of other basidiomycetes.

## Introduction

*Ganoderma lucidum* is one of the most widely cultivated pharmaceutical mushrooms in East Asia because it contains many pharmacologically active compounds, such as ganoderic acid and polysaccharide [1]–[3]. In recent years, many studies have described the pharmacological activity and chemical composition of *G. lucidum* [4]–[6]. These pharmacological compounds, which inhibit tumor cell growth [7], modulate the immune system and lower blood sugar levels [8], are thought to be potential candidates for drug discovery, and the pharmacological value of *G. lucidum* has drawn widespread interest [9].

The number of studies investigating the molecular genetics of *G. lucidum* has increased and includes genome analysis [10], [11], gene cloning and the characterization of biosynthetic pathways [12]–[17], cloning of other functional genes [18]–[20], and EST analysis [16], [21]. However, further functional analysis of genes of interest has been limited due to the lack of effective genetic tools. *G. lucidum* has been considered a genetically intractable organism, and tools for investigating the functional genomics of *G. lucidum* are still at an early stage, although some gene transfer procedures have been established [22]–[24]. The roles of the genes within the biosynthesis network are not yet clear. Improving the genetic tools for this organism will provide significant insight into the mechanism of synthesis of pharmacologically active compounds, open new possibilities for the exploitation of *G. lucidum* for enhancing pharmacologically active compounds by genetic engineering, and be critical for studying the biology, chemical composition, and evolution of this organism.

Reverse genetic methods, such as gene disruption or RNA interference, can be useful for studying functional genomics [25], [26]. RNA interference (RNAi) is a convenient reverse genetic method that has been widely used in several types of organisms [27], [28] because it is simple to down-regulate the target gene using short DNA sequences, and the use of RNAi circumvents the low homologous recombination rate that occurs with gene disruption. RNAi uses sequence-specific double-stranded RNA (dsRNA) to trigger the degradation of homologous mRNA, thereby reducing gene expression.

Among filamentous fungi, RNAi was first reported in *Neurospora crassa* [29], and this method has been harnessed to elucidate gene function in an expanding number of organisms [30]–[32]. However, the basidiomycetes group lacks these RNAi methods [33], except for some model species, such as *Schizophyllum commune* [34], *Coprinopsis cinerea* [35], *Laccaria bicolor* [36], and several cultured mushrooms, such as *Agaricus bisporus* [37], *Lentinula edodes* [24], and *Clitopilus passeckerianus* [22]. Although these studies reported wide variation in the frequency of silenced transformants, no clear information exists about the optimal method for silencing a gene in basidiomycetes because the previous studies used different target genes and vector configurations. In *G. lucidum*, it was unknown which method could induce silencing and which could yield the highest efficiency of silencing. Therefore, the discovery and development of a convenient and efficient RNAi system are important areas for further study in *G. lucidum* and other basidiomycetes.

In this study, the endogenous orotidine 5′-monophosphate decarboxylase gene (*URA3*) was cloned as a reporter for monitoring RNAi in *G. lucidum*, and a convenient RNAi system was established through the development of a simple electroporation procedure. The effectiveness of four different RNAi methods was analyzed, and the dual promoter silencing method was determined to yield the highest fraction of *URA3*-silenced transformants. Finally, we succeeded in using this dual promoter silencing system to co-silence two endogenous genes in *G. lucidum* and demonstrated the applicability of this RNAi system.

## Results

### Isolation of genomic DNA and cDNA of *URA3* in *G. lucidum*

A 400-bp PCR product was amplified using the degenerate primers URA3-1 and URA3-2 (table 1), which were designed based on the conserved regions of the *URA3* gene sequence. The 5′ self-formed adaptor PCR (SEFA) and 3′ rapid-amplification of cDNA ends (RACE) methods were utilized to obtain the full-length sequence of the gene. The full-length *URA3* genomic sequence was 947 bp in length, including two introns. The full-length *URA3* cDNA contained an 840 bp ORF. A BLASTX (http://www.ncbi.nlm.nih.gov/) search confirmed that the sequence was highly homologous to the *URA3* genes of *Schizophyllum commune* (73% identity, 83% homology), which indicated that the amplified sequence was indeed from the *G. lucidum* URA3 gene. The nucleotide sequences of the *URA3* gene and cDNA have been deposited in the GenBank database under accession numbers JQ406674 and JQ406675.

**Table 1:** Oligonucleotide primers used.

Primer	Sequence (5′ to 3′)*	Description
URA3-1	AARTTYGCNGAYATHGGNAA	Degenerate primers amplify a specific fragment of *URA3*
URA3-2	GGNGTVCGRTAYTGYTGNCC	
URA3RACE1	TAAGATGTAGTCCCTTTGCG	Get the 3′-end of *URA3*-cDNA
URA3RACE2	CGCCGAGATGAGCACCAAG	
URA3-5Sp1	GCAGTATCCGACGGCATCAGCATTCTATCTC	Amplify the 5′-end of *URA3*-DNA
URA3-5Sp2	TACAACCTGCGTATCCTTATGCTCCACCCAC	
URA3-5Sp3	GCCTTATCAGACTCAANNNNNNNNNATGTAG	
URA3-F	ATGGTGGCCGTGGCCAAGC	Amplify the whole length
URA3-R	CTAATCCGAGATCCCAACCC	
hp-Anti 1	GCGCGGTACCTCTCCGCCTGTGCGCGGACT	A antisense fragment of URA3 for the pAN7-ura3-hp plasmid
hp-Anti 2	TCTCCTCGAGACGATCGAGCGCAAGCGCAC	
hp-Sense 1	ATCGCTCGAGGGTGGCACTTCAATATTCTG	A sense fragment of URA3 for the pAN7-ura3-hp plasmid
hp-Sense 2	GCGCAAGCTTTCTCCGCCTGTGCGCGGACT	
hp-gpd 1	TCGAGGATCCGGTATATCGTTACATATATC	The *G. lucidum* gpd promoter for the pAN7-ura3-hp plasmid
hp-gpd 2	CGATGGTACCAGGGGGATGAAGAGTGAGTA	
S-Sense 1	GCGCGGTACCGGTGGCACTTCAATATTCTG	The sense fragment of URA3 for the pAN7-ura3-s plasmid
S-Sense 2	GCGCAAGCTTTCTCCGCCTGTGCGCGGACT	
AS-Anti 1	GCGCGGTACCTCTCCGCCTGTGCGCGGACT	The antisense fragment of URA3 for the pAN7-ura3-as plasmid
AS-Anti 2	GCGCAAGCTTGGTGGCACTTCAATATTCTG	
Dual-35S1	TCATCTAGAAGAGATAGATTTGTAGAGAG	The 35s promoter for the pAN7-ura3-dual plasmid
Dual-35s2	GATAAGCTTCGTAATCATGGTCATAGCTG	
URA3-real1	TGCCGACATTGGAAACACG	Detects the *URA3* expression
URA3-real2	TTGCGAGGCTGCCCTTGGT	
Lcc-1	AGTCCGCGCACAGGCGGAGAATGGTGAAATTCCAATCGTT	Overlap PCR amplify the *laccase* and *URA3* fragment
Lcc-2	TCTAGACACCTTGTTCCCTGTGATGA	
URA-LCC	AACGATTGGAATTTCACCATTCTCCGCCTGTGCGCGGACT	
Lcc-real 1	CACACAATGCTGAAGACCAC	Detects the *Laccase* expression
Lcc-real 2	CGGAACCTGGAAATCGTAGA	
Hph 4	TCGTTATGTTTATCGGCACTTT	Detects the transformants' selectable marker hph (hygromycin phosphotransferase) gene
Hph 5	GATGTTGGCGACCTCGTATT	

* R = A, G; Y = T, C; N = A, T, C or G; H = A, T or C; V = A, C or G

### Transformation of *G. lucidum*

In an attempt to detect whether pGL-GPE, which contained the hygromycin phosphotransferase (*hph*) and enhanced green fluorescence protein (*egfp*) genes, could be transferred into *G. lucidum*, the transformants were selected on CYM medium containing 100 µg/mL hygromycin B. Colonies appeared after 7 days, and the transformation efficiency was approximately 30 transformants per µg of plasmid pGL-GPE DNA [31] (data not shown). In addition, the use of higher resistance and field strength may have increased the transformation efficiency; however, the chance of cuvette failure would also have increased.

PCR analysis was performed to detect the presence of the fusion fragment containing the *hph* gene in genomic DNA isolated from the hygromycin B-resistant transformants. The *hph* gene was detected in 23 of 34 transformants (data not shown). To determine whether the putative transformants could stably maintain hygromycin B resistance, 10 randomly selected transformants were grown on CYM plates without hygromycin B and re-plated for 3 months. We recovered 8 mitotically stable transformants. These results indicate that the transformants can maintain the desired phenotypes long enough for further study.

**Figure 1:**
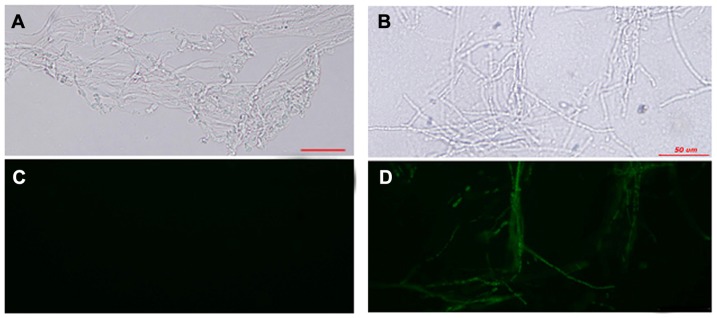
Expression of GFP in *G. lucidum*. A GFP-expressing *G. lucidum* transformant was cultivated on CYM agar medium at 28°C and examined microscopically. Fluorescence of wild-type *G. lucidum* (A and C) and the GFP transformant (B and D) was examined. Mycelia were removed from actively growing colonies, suspended in sterile water, and viewed microscopically. Samples shown in panels A and B were examined using bright-field microscopy, and samples in panels C and D were viewed using blue-field microscopy. Bars = 50 µm.

To determine whether exogenous genes were expressed in the pGL-GP transformants, the expression of GFP in transformants cultured on CYM plates without hygromycin B and re-plated for 10 days was detected by fluorescence imaging (Fig. 1). While no GFP fluorescence was detected in wild-type mycelia, the transformants exhibited a positive GFP signal. These results indicate that the method is reliable for the transformation of *G. lucidum*.

### Presence of a Dicer-1 homologue in *G. lucidum*

Genome sequence alignment, which was supplied by Shilin Chen and Haibin Xu (The Institute of Medicinal Plant Development, Chinese Academy of Medical Sciences), was used to identify a Dicer-1 homolog in the *G. lucidum* genome. Fig. S1 shows the conserved domains detected in this protein fragment using the NCBI Conserved Domain Database. Sequence analysis identified 4 domains characteristic of DCL proteins: a DEAD domain, a helicase C domain, a dsRNA-binding domain and two RNase 3 domains. This sequence is of a 4635 bp fragment that encodes 1544 amino acids, corresponding to a central internal fragment of a Dicer-1 protein homolog (Fig. S2). The Panther Classification System identified this protein as Dicer-1 with an E value of 6.5 e^−73^.

Fig. S3 shows the amino acid sequence alignment of the GLDCL-1 fragment to other fungal DCL homologues. This alignment demonstrates that these proteins are conserved among fungi, specifically in the regions of the previously mentioned domains.

### Construction of RNAi vectors

**Figure 2:**
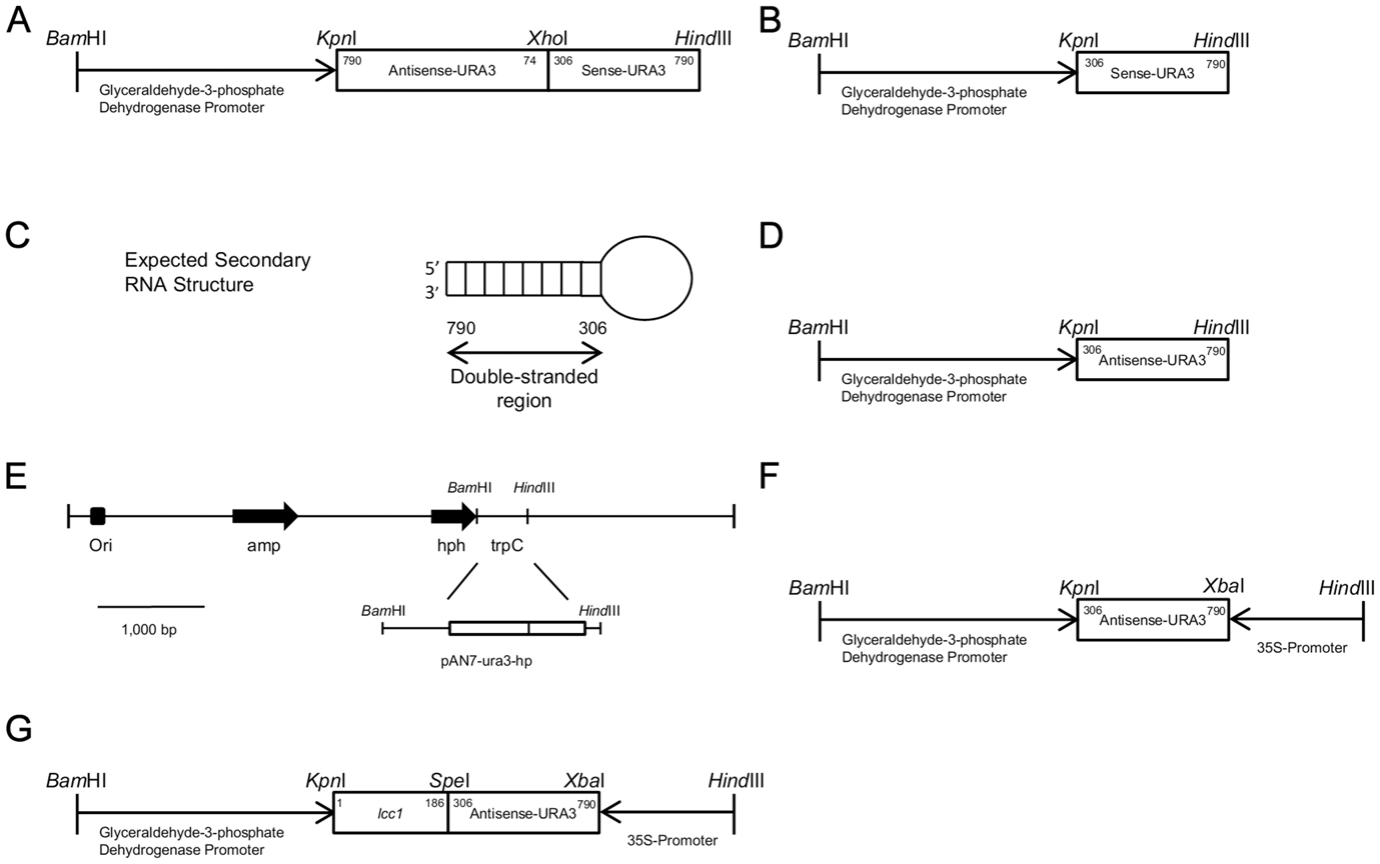
Construction of the *URA3* RNAi expression cassette plasmids used to silence *URA3*. In all plasmids except for the dual promoter plasmid, *URA3* transcription is driven by the glyceraldehyde-3-phosphate dehydrogenase promoter. pAN7-ura3-hp contains the antisense fragment of *URA3* from 790-74 bp and the sense fragment from 306-790 bp (A). pAN7-ura3-s contains 484 bp of *URA3* in the sense orientation (B). Complementary portions of the antisense and sense regions are paired in the 306-790 nucleotide region of the *URA3* gene, forming a hairpin loop with a 484 bp complementary region (C). pAN7-ura3-as contains *URA3* in the antisense orientation (D). Maps of the pAN7-1 vector and the inserted RNAi cassettes (E). The dual promoter plasmid contains 484 bp of *URA3* between the gpd promoter and 35S promoter (F). The ura3-lcc co-silencing vector used the dual promoter method (G). Bar = 1000 bp

To examine the variables associated with silencing, four vectors were generated that expressed a 484 bp fragment of the *URA3* gene in hairpin, sense, antisense, and dual promoter constructs, named pAN7-ura3-hp, pAN7-ura3-s, pAN7-ura3-as, and pAN7-ura3-dual, respectively (Fig. 2).

The pAN7-ura3-hp plasmid was expected to encode a hairpin RNA comprised of two 484 bp complementary regions separated by a 232 bp spacer fragment (Fig. 2A). The pAN7-ura3-hp RNA transcript structure is typical of many functional RNAi products, and the 484 bp complementary stem region is sufficiently long to silence the target gene [32]. The remainder of the antisense fragment forms a loop (Fig. 2C), which contributes to the stability of the inverted repeat sequence [38]. pAN7-ura3-s and pAN7-ura3-as contain the 484 bp fragment of *URA3* from position 306-790 (Fig. 2B; Fig. 2D). The pAN7-ura3-dual plasmid was constructed with the 484 bp fragment of *URA3* and two promoters: Pgpd (*gpd* promoter) and P35s (*35s* promoter) (Fig. 2F). Maps of the pAN7-1 vector and the inserted RNAi cassettes are shown in Fig. 2E.

### Selection of RNAi transformants and phenotypic analysis

**Figure 3:**
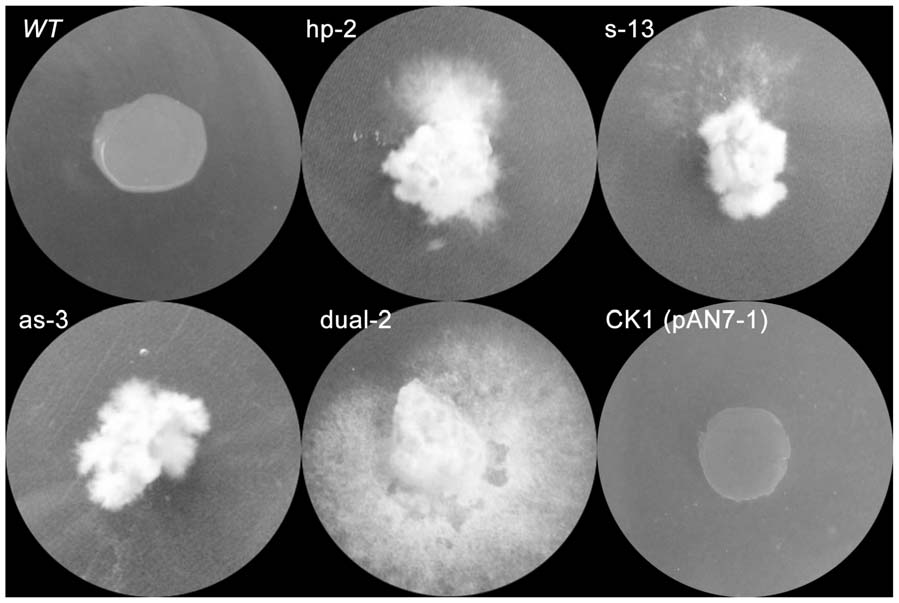
Phenotypic assays of the RNAi transformants on 5-FOA plates. *URA3*-silenced transformants, wild-type (*wt*) and control transformants (CK1, transformed with plasmid pAN7-1) were cultured on CYM medium containing 600 µg/mL 5-FOA for ten days. Hp-2 was one of the pAN7-ura3-hp transformants, s-13 was one of the pAN7-ura3-s transformants, as-3 was one of the pAN7-ura3-as transformants, and dual-2 was one of the pAN7-ura3-dual transformants.

The four silencing plasmids (pAN7-ura3-hp, pAN7-ura3-s, pAN7-ura3-as, and pAN7-ura3-dual) were introduced by electroporation into *G. lucidum*. To reduce the false positive rate, all of the hygromycin B-resistant transformants were subcultured on CYM medium without selection for 6 generations (approximately 3 months) and then re-cultured on CYM with 600 µg/mL 5-FOA (5-fluoroorotic acid) to select for the silenced transformants. Fig. 3 shows that different transformants and the *wt* strain exhibited different levels of 5-FOA tolerance. The *ck1* strain, which contains the empty vector (pAN7-1) as a control, exhibited the same phenotype as the *wt* strain. The *wt* strain and the *ck1* strain did not grow or form colonies on CYM containing 600 µg/mL 5-FOA, whereas the *URA3*-silenced transformants did grow after approximately two weeks of culturing. These results demonstrate that the RNAi remained stable across mitotic cell divisions for at least 3 months. However, not all of the transformants obtained stable silencing or could grow on CYM containing 600 µg/mL 5-FOA (data not shown).

Transformants were categorized as silenced and non-silenced based on their tolerance to 5-FOA: the representative transformants could be cultured on CYM containing 600 µg/mL 5-FOA, whereas the mycelia of non-silenced transformants could not germinate on this selective medium (table 2; Fig. 3). The effectiveness of the silencing was determined in 202 transformants. Both silenced and non-silenced transformants were generated with all four of the silencing plasmids and an empty vector (pAN7-1). The dual promoter silencing method had the highest silencing efficiency, with a mean silencing value of 81.9%; in addition, the dual promoter method allows single-step non-oriented cloning for further vector construction (Table 2). All 40 of the *ck1* transformants exhibited the non-silenced phenotype (Table 2). This indicates that all four of the RNAi methods can produce the silenced phenotype and that the dual promoter silencing method has the highest silencing efficiency in *G. lucidum*. The control results demonstrate that the silenced phenotype was not caused by the transfer procedures.

**Table 2:** Number of transformants with silenced and no-silenced.

Plasmid Configuration	Construction steps for further study	Replicate	silenced	non-silenced
Hairpin (pAN7-ura3-hp)	2–3	I	7	4
		II	8	7
		III	9	5
		Mean(%)	60.4	39.6
Sense (pAN7-ura3-s)	1	I	8	5
		II	6	11
		III	8	3
		Mean(%)	56.5	43.5
Antisense (pAN7-ura3-as)	1	I	11	7
		II	8	6
		III	4	7
		Mean(%)	51.5	48.5
Dual-promoter (pAN7-ura3-dual)	1	I	10	0
		II	19	5
		III	16	8
		Mean(%)	81.9	18.1
CK1 (pAN7-1)	0	I	0	11
		II	0	15
		III	0	14
		Mean(%)	0	100

**Figure 4:**
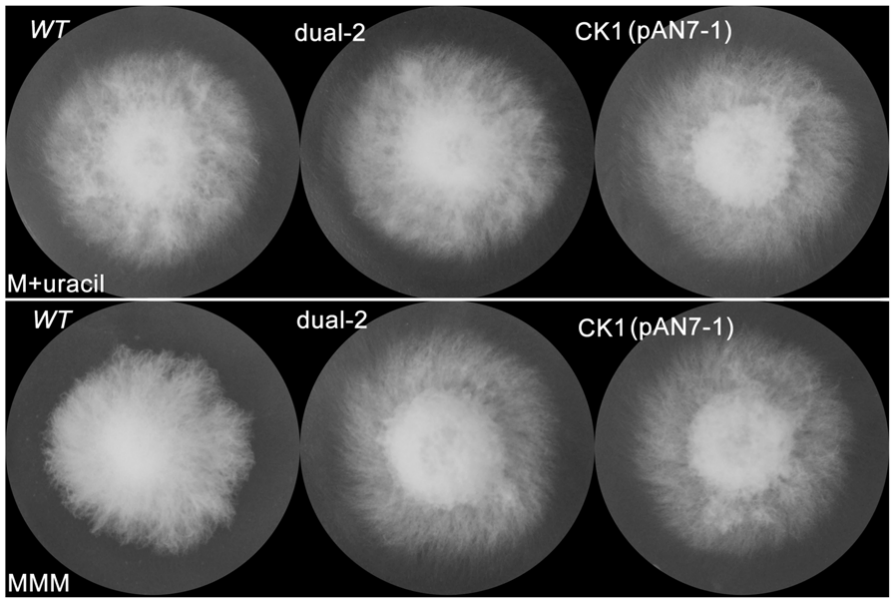
Phenotypic assays of the RNAi transformants on MMM plates. *URA3*-silenced transformants (dual-2), wild-type (*wt*) and control transformants (CK1, transformed with the pAN7-1 plasmid) were cultured on MMM medium with or without uracil (100 mg/L) for 7 days at 28°C. The upper row (M+uracil) is MMM medium with uracil, and the lower row (MMM) is MMM medium.

If *URA3* were fully silenced, the silenced transformants would be expected to behave as orotidine 5′-monophosphate decarboxylase (OMPdecase) auxotrophs and be unable to grow on mushroom minimal medium (MMM, which is commonly used for detecting auxotrophic strains of mushrooms). However, the growth rate of the RNAi transformants did not differ from that of the *wt* strain or the *ck1* transformant on MMM with or without uracil (100 mg/L) (Fig. 4). This result is the same as was obtained with *Agaricus bisporus* [37].

### Quantification of *URA3* expression in RNAi transformants using the four RNAi methods

**Figure 5:**
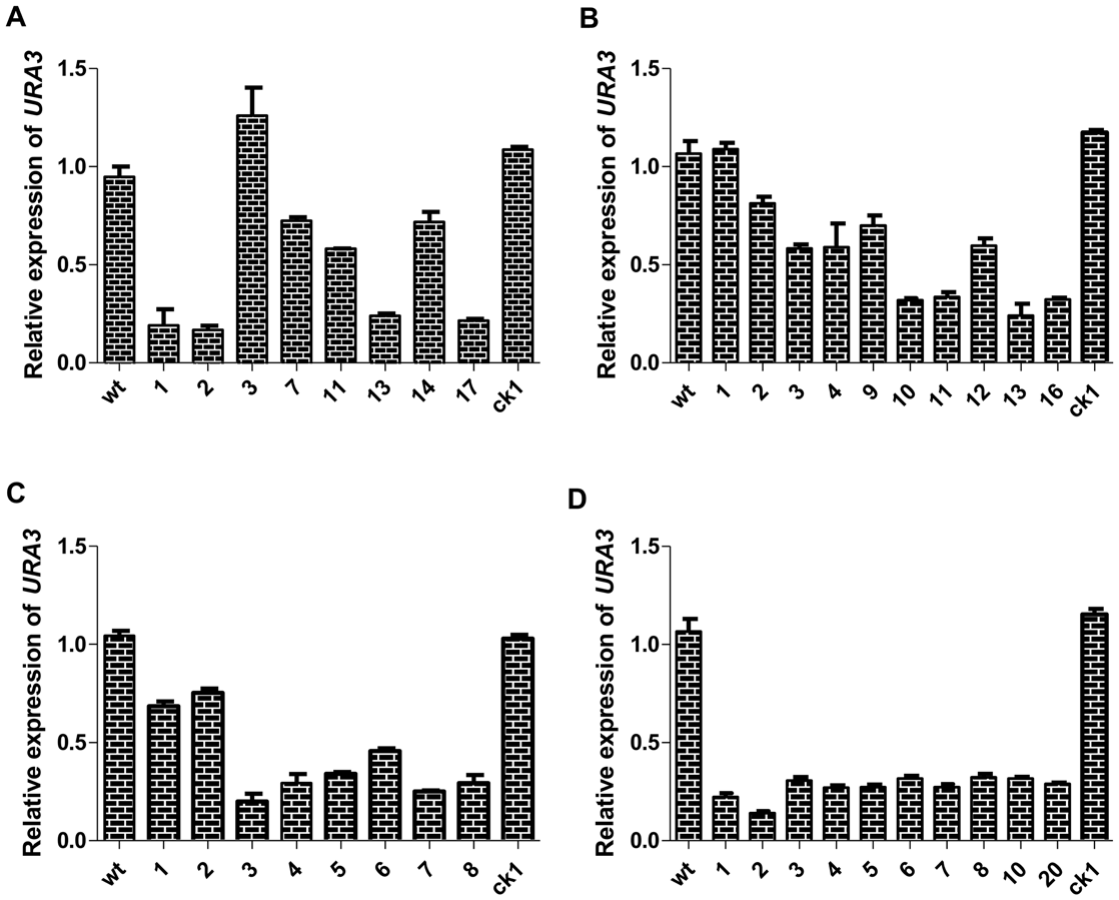
Measurement of *URA3* gene expression as determined by real-time PCR. Real-time PCR analysis was performed on pAN7-ura3-hp (A), pAN7-ura3-s (B), pAN7-ura3-as (C) and pAN7-ura3-dual (D) transformants. The relative mRNA levels of *URA3* were calculated as the ratio of *G. lucidum URA3* mRNA to endogenous *18S* rRNA. The upper running title of each panel is the ratio of *URA3* mRNA and *18S* rRNA mRNA compared with *wt*. The lower running title of each panel is the silenced transformant, *wt* and *ck1* strains. Wild type was considered to have a value of 1.0 for all experiments trials because the mRNA expression in all other isolates was relative to wt. Values are the means ± SE (n = 3).

To examine the RNAi transformants on the molecular level, transcription in the *URA3*-silenced transformants was examined by real-time PCR. The *URA3* transcription levels were normalized to those of the endogenous *18S* rRNA gene. Transformants were randomly chosen from each RNAi method to quantify the reduction in mRNA transcript levels at this locus. All four methods downregulated the expression of *URA3*, albeit to varying degrees (Fig. 5).

Among the selected pAN7-ura3-hp transformants, 4 of 8 exhibited significant suppression of *URA3*, with relative expression reduced 70% compared with the *wt* and *ck1* strains (Fig. 5A). The pAN7-ura3-s and pAN7-ura3-as transformants exhibited results similar to the pAN7-ura3-hp transformants, with a reduction in the relative expression of *URA3* ranging from 30% to 75% (Fig. 5B; Fig. 5C). The mRNA transcripts of the ten randomly selected pAN7-ura3-dual silenced transformants were downregulated, and our results show that all ten clones appeared to have achieved a fairly high efficiency of *URA3* knockdown compared with the *wt* or *ck1* strains (Fig. 5D). These results also indicate that the dual promoter silencing vector yields the highest rate of *URA3* silencing.

### Multiple genes can be co-silenced using the dual promoter silencing vector

Given that the silencing of *URA3* could be selected with 5-FOA, we constructed a co-silencing vector (pAN7-lcc1-ura3-dual) using *URA3* as a selectable marker to facilitate the silencing of other genes (Fig. 2G). The co-silencing transformants would exhibit both the *URA3-* and *lcc1-* silenced phenotypes when the two genes were both silenced. To determine the silencing efficiency of the transformants, 30 of 36 hygromycin B-resistant transformants were selected on CYM medium containing 600 µg/mL 5-FOA. Then, 10 of the phenotypically 5-FOA-positive transformants were randomly chosen for further study.

**Figure 6:**
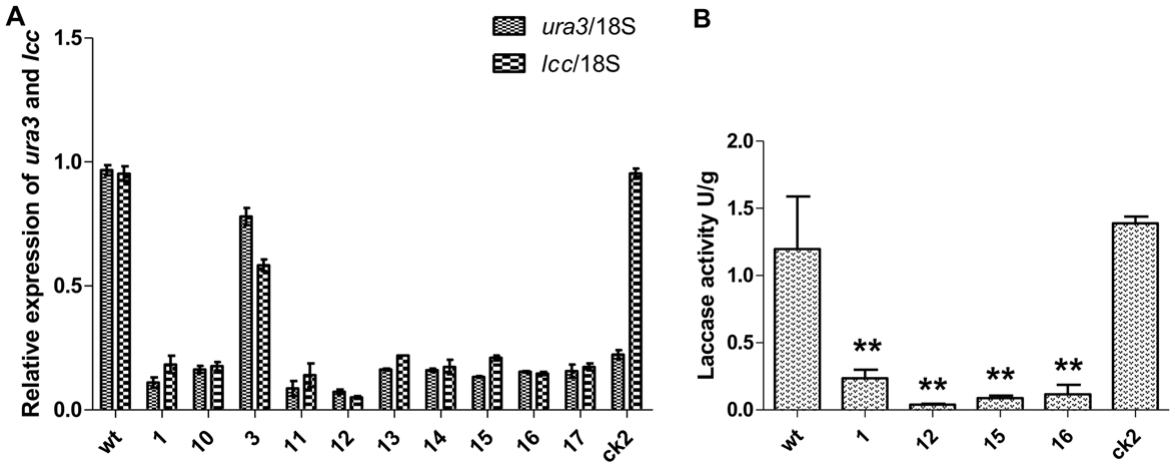
Measurement of co-silenced gene expression as determined by real-time PCR and laccase enzyme assays. (A) Real-time PCR analysis was performed in dual promoter co-silenced transformants. The relative mRNA levels of *URA3* and *lcc1* were calculated as the ratio of *URA3* and *lcc1* mRNA to endogenous *18S* rRNA. The upper running title of each panel is the ratio of target genes and *18S* rRNA mRNA compared with *wt*. The lower running title of each panel is the silenced transformant, *wt* and *ck2* strains. The dotted column is *ura3*/*18s*, and the black rectangle column is *lcc1*/*18s*. (B) Laccase activity was monitored in the filtrate cultures using an ABTS oxidization test. Wt represents the wild-type strain, *ck2* strains contain the pAN7-ura3-dual vector, and strains 1, 12, 15, and 16 represent ura3-lcc co-silenced transformants. Values are means ± SE (n = 3). Asterisks represent significant differences among the strains according to a one-way ANOVA test (P<0.01).

**Figure 7:**
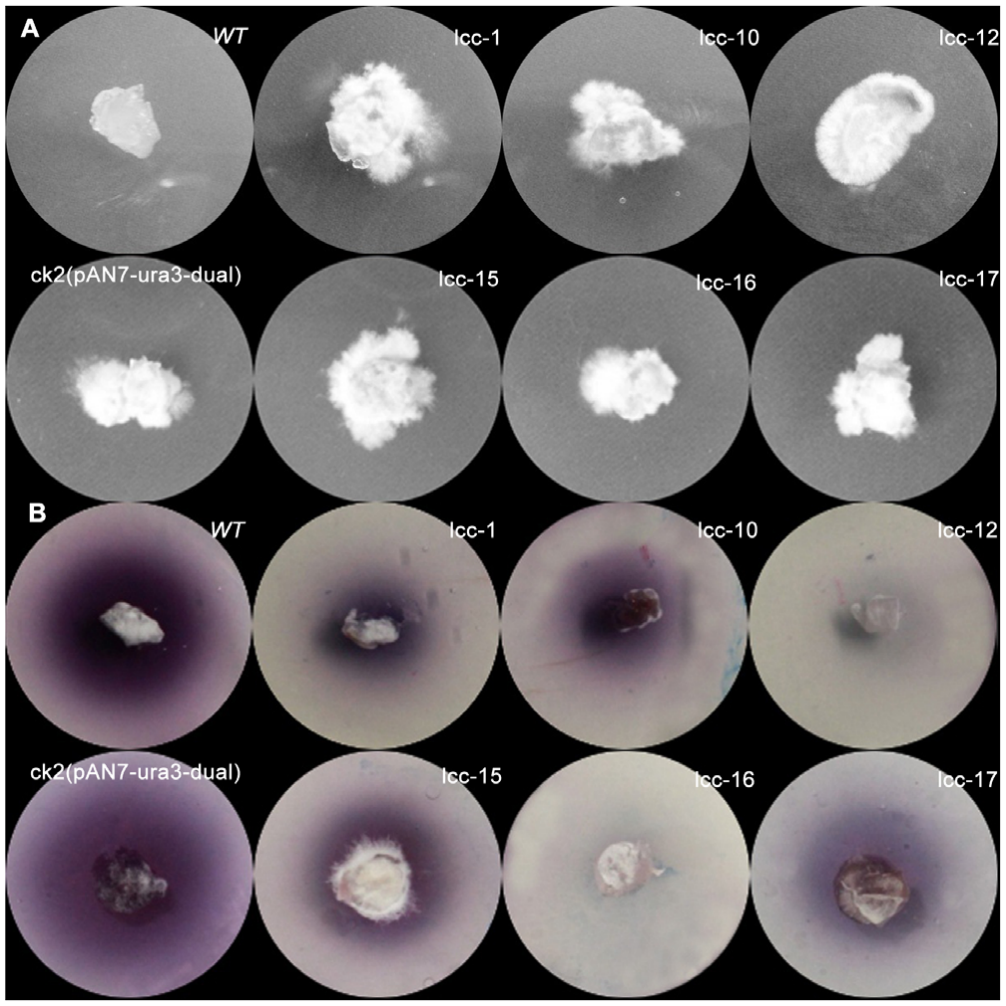
Phenotypic assays of co-silenced transformants. (A) *URA3-lcc1* co-silenced transformants, wild-type and *ck2* transformants were cultured on CYM medium containing 600 µg/mL 5-FOA for ten days. Wt represents the wild-type strain, *ck2* strains contain the pAN7-ura3-dual vector, and lcc-1, 10, 12, 15, 16, and 17 represent ura3-lcc co-silenced transformants. (B) An ABTS-Petri dish was used to determine whether *laccase* was silenced in the co-silencing transformants. Lcc-1, lcc-10 lcc-12, lcc-15, lcc-16, and lcc-17 had significantly smaller colored circles compared with the *wt* and control strains.

Real-time PCR analysis of the *URA3* and *lcc1* transcripts demonstrated that the *URA3* and *lcc1* transcripts were downregulated in most of the transformants compared with the *wt* strain. The *ck2* strains containing the pAN7-ura3-dual plasmid exhibited no difference in *lcc1* expression compared with *wt*, whereas *lcc1* expression in co-silenced transformants was reduced. Furthermore, the reduction in levels of the *URA3* and *lcc1* mRNAs was correlated (Fig. 6A). The silencing of laccase was examined using ABTS-Petri dishes (2,2′-azino-di-3-ethylbenzothiazoline-6-sulfonate, Sigma), in which the chromogen ABTS is a very sensitive substrate that allows the rapid screening of fungal strains that produce extracellular laccases by means of a chromogenic reaction. All of the randomly selected co-silenced transformants that were 5-FOA-tolerant (Fig. 7A) had significantly fewer colored circles than the *wt* and *ck2* strains (Fig. 7B). In two of the *URA3-LCC* co-silenced transformants (lcc-12, lcc-16), the color change was barely detectable. Upon further analysis, the laccase assay, which was measured by monitoring the increase in absorbance at 420 nm using ABTS as a substrate, demonstrated that the enzymatic activity of RNAi transformants was greatly reduced when compared with *wt* or *ck2* (Fig. 6B). Laccase activity was consistent with the phenotypes (Fig. 7B). These results indicate that it is possible to use *URA3* as a reporter for co-silencing another gene, and the control results with *ck2* demonstrate that co-silencing was not caused by the transfer procedures.

## Discussion

In this study, we have shown that the transformation procedure based on the electroporation of mycelial fragments is a convenient method for introducing transgenes into *G. lucidum* because it obviates the complicated production of protoplasts in previously described methods [39] and results in hygromycin B resistance and GFP expression in the transformants. Using this method, we succeeded in silencing the endogenous *URA3* gene and co-silencing *lcc1* and *URA3* by reducing their mRNA and enzyme levels. Because of the uncertainty regarding the presence of a gene-silencing mechanism in some fungi, such as *Saccharomyces cerevisiae* and *Ustilago maydis* [40], we identified the presence of a Dicer-1 homolog in the *G. lucidum* genome (Fig. S1, Fig. S2, Fig. S3). The Dicer enzymes are important components of the mechanism that processes double-stranded RNA precursors into small RNAs during gene silencing [41], [42], and the identification of this homolog implies that *G. lucidum* can use RNAi for gene silencing, which is also supported by our results.

Although most RNAi studies performed in filamentous fungi report the use of sense, antisense, dual promoter, or hairpin constructs, previous publications did not directly compare the efficiency of these silencing constructs in filamentous fungi. To systematically examine vectors associated with silencing in *G. lucidum*, four RNAi plasmids carrying the silencing fragment of the same gene were transferred into this species. Our data unequivocally indicate that a dual promoter silencing vector yields the highest rate of *URA3* silencing (Table 2). The independent transcription of a target gene from each promoter may produce a pool of sense and antisense RNAs in the cell that combine to form long dsRNAs, which can then be processed into small interfering RNAs (siRNAs) by Dicer [43]. These dual promoter transformants appear to produce much more uniform results than other vectors (Fig. 5D, Fig. 6A) and make it possible to select silenced transformants by fewer tests. Our data suggest that the dual promoter silencing system may be a powerful tool for loss-of-function analysis in *G. lucidum*. In addition to the high silencing efficiency, the greatest advantage of the dual promoter system is that it allows single-step non-oriented cloning for vector construction (table 2). This advantage allows not only fewer cloning steps but also the construction of multiple silencing vectors in a single transformation experiment. To highlight this advantage, we constructed a co-silencing system using mixed PCR (Fig. 2G) and were able to co-silence two genes simultaneously. When cDNA inserts are made from a normalized mRNA pool, a silencing library consisting of thousands of genes may be constructed in a single transformation [12].

However, the silencing induced by dual promoter as well as other RNAi systems was not always absolute. Some of the transformants did not grow on 5-FOA medium (data not shown), or the silencing levels were different between transformants. These results were consistent with those of our predecessors [34], [37]. The phenotypic variability among RNAi transformants may be related to the condition of the RNAi cassettes; for example, the site of transgene integration, which influences the expression level of the silencing RNA, affects the silencing level [25]. In addition, the copy number of the RNAi cassettes would also affect the silencing level of independent transformants [27]. Furthermore, the variable activity of the RNA-dependent RNA polymerase (RdRP) in different transformants would also affect the silencing level between independent transformants because the as RdRP can extend dsRNA synthesis from the antisense primer into the regions upstream of the target gene [27]. The incomplete silencing of the *URA3* locus observed in our study is not an unusual result; indeed, many species of fungi have shown incomplete gene suppression by RNAi [24], [36], [44], although most gene functions can be studied only with preselected strains [45]. To overcome these difficulties, several previous publications have used vector systems in which a reporter gene is downregulated simultaneously with an endogenous target gene. Such RNAi systems use either strain-specific marker genes that can be identified phenotypically [46] or, alternatively, a generally applicable exogenous reporter gene, such as GFP or red fluorescent protein (DsRed) [33], [45]. However, these RNAi systems are strain-specific or require the construction of an exogenous gene expression strain with multiple resistance markers. In this report, endogenous *URA3*, which encodes OMPdecase and catalyzes the conversion of orotidylic acid to ruidylic acid during uracil biosynthesis in fungi, was cloned and silenced The OMPdecase of fungi can convert 5-FOA into the toxic compound 5-fluorouracil (a suicide inhibitor) causing death (negative selection) [22]. Our results show that the *URA3*-silenced transformants could be selected on 5-FOA medium (Table 2; Fig. 3) and this phenotype was easy to detect, so we used this characteristic to construct a co-silencing RNAi system containing this gene as a reporter gene. To monitor the efficiency of the co-silencing system, *URA3* was co-silenced with *Lcc1*, which encodes one of the *laccase* genes in *G. lucidum* and can be easily detected using ABTS [47]. All of the randomly selected *ura3-lcc1* co-silenced transformants, which were selected after the 5-FOA assay, exhibited a significant reduction in mRNA levels and enzymatic activity of laccase, and the reduction in levels of the mRNA of *lcc1* correlated with those of *URA3* (Fig. 6; Fig. 7). Therefore, co-silencing *URA3* could improve the monitoring efficiency of RNAi for another gene linked with *URA3*, and constructing other co-silencing victors by replacing *lcc1* would be straightforward. The *lcc1*-silenced transformants will help us to better understand its function in *G. lucidum* in the future.

In addition to the advantages of RNAi, these technique also has limitations, which have been mentioned in many reports [32], [48], and some of these limitations were also observed in this study. The disadvantages primarily include inconsistency due to incomplete and/or reversible silencing [49] and variations in the silencing specificity of different genes. The latter shortcoming can actually be harnessed as an additional advantage, especially when silencing of multiple genes is required [48]. Due to incomplete or reversible silencing, RNAi is not preferred for studying non-essential gene functions compared with gene deletion or knockout.

*URA3* mutant strains cannot grow on minimal medium without uracil [22]. However, in our study, there was no difference between the growth rate of RNAi transformants and the *wt* strain or the *ck1* transformant on MMM medium with and without uracil. This result was the same as that observed in *Agaricus bisporus* [37]. Similarly, the use of antisense constructs to suppress another uracil biosynthesis gene, *PYR3* (dihydroorotase), in the heterobasidiomycete *Ustilago maydis* failed to yield mutant phenotypes [50]. Collectively, these results suggest that although substantial downregulation of mRNAs can be achieved through RNAi transformations, very low-level transcription of key biosynthetic genes may be sufficient to permit the growth of the organism on selective media. However, *URA3* can be used as a selectable marker using 5-FOA medium.

The molecular tools that we have described in this paper may aid in identifying and manipulating the genes of this pharmaceutically important species. More importantly, this co-silencing system will make it possible for us to silence genes in a high-throughput manner, and these tools should also facilitate studies aimed at gene isolation or characterization in other mushrooms.

## Materials and Methods

### Strains and culture conditions

The DH5α strain of *Escherichia coli* was used for plasmid amplification and was grown in Luria–Bertani (LB) medium containing 100 µg/mL ampicillin or 50 µg/mL kanamycin as required. *G. lucidum* strain HG was grown at 28°C in CYM medium (1% maltose, 2% glucose, 0.2% yeast extract, 0.2% tryptone, 0.05% MgSO_4_·7H_2_O, 0.46% KH_2_PO_4_) or MMM medium (2% glucose, 0.2% DL-asparagine, 0.05% MgSO_4_·7H_2_O, 0.046% KH_2_PO_4_, 0.1%K_2_HPO_4_, 120 µg/L thiamin-HCl) and was used as the recipient host strain for transformation and gene silencing.

### Amplification of the full-length sequence of *URA3*

Based on the highly conserved amino acid regions of known orotidine 5′-monophosphate decarboxylase genes (*URA3*) from other fungi, two degenerate primers, URA3-1 and URA3-2 (Table 1), were synthesized for the amplification of a specific 400 bp fragment of the *URA3* gene. A BLASTX search confirmed that the specific fragment of the *G. lucidum URA3* gene was highly homologous with other fungal *URA3* genes. Subsequently, this sequence was used to design and synthesize gene-specific primers to clone the full-length sequences. Rapid amplification of cDNA ends (RACE-PCR) was performed to obtain the sequence of the 3′ end of the cDNA using the specific primers URA3-RACE1 and URA3-RACE2 (Table 1). Self-formed adaptor PCR (SEFA-PCR) [51] was performed to amplify the 5′ end of the DNA and promoter region using three gene-specific primers (URA3-5Sp1, URA3-5Sp2, URA3-5Sp3) (Table 1). By aligning and assembling the sequences of the 3′ RACE and 5′ SEFA products and the specific fragment, the full-length sequence of *URA3* was deduced and subsequently amplified by PCR using the primers URA3-F and URA3-R (Table 1). The amplified product was purified, cloned into the pMD18-T vector (TARAKA, Dalian, China), and sequenced.

### Construction of RNA interference cassettes for *URA3* and *lcc1*

RNAi cassettes were designed according to the *URA3* gene of *G. lucidum* (GenBank accession number JQ406675). Expression cassettes were designed to downregulate the expression of the *URA3* gene of *G. lucidum* strain HG. Constructs incorporated the 484nt *URA3* coding region in the expression plasmid pAN7-1 [52] (Fig. 2). The hairpin plasmid, which was defined as pAN7-ura3-hp, was constructed by amplifying a 716 bp antisense and a 484 bp sense fragment of the *URA3* gene using the primers shown in table 1. Expression of the linked antisense/sense *URA3* gene fragments was driven by the glyceraldehyde-3-phosphate dehydrogenase (*gpd*) promoter (GenBank: DQ404345.1) [10] (Fig. 2A). The *gpd* promoter, antisense *URA3* and sense *URA3* fragments were sequentially ligated into the pAN7-1 vector by replacing the *TrpC* terminator. The sense plasmid, called pAN7-ura3-s, was produced by amplifying the 484 bp sense fragment flanked with the restriction sites *Kpn*I and *Bam*HI and inserting it into the plasmid pAN7-ura3-hp after *Kpn*I/*Hind*III restriction digestion (Fig. 2B). The antisense vector pAN7-ura3-as was constructed using the same method (Fig. 2D). The dual promoter plasmid pAN7-ura3-dual was generated by the amplification of three DNA fragments: the *gpd* promoter (Pgpd), the antisense fragment and the *35S* promoter (P35S) (table 1). The *35S* promoter was amplified using the plasmid pCAMBIA-1300 (CAMBIA, Canberra) as the template (table 1) and was subsequently cloned into pAN7-1 (Fig. 2F). The *URA3* and *lcc1* co-silencing cassettes (GenBank: FJ656307.1) were produced by mixed PCR, cloned into pAN7-ura3-dual and renamed pAN7-lcc1-ura3-dual, and this vector contained the RNAi cassettes of *URA3* linked with *lcc1* (Fig. 2G). The constructs were verified by nucleotide sequence analysis to confirm the presence of all of the required elements of a functional cassette prior to their transformation.

### Transformation procedure

All plasmids were transferred into *G. lucidum* by electroporation [39] with some modifications. The procedure and electrical pulse delivery test conditions included several settings [53], and the appropriate modifications were as follows:

First, four-day-old liquid cultures of *G. lucidum* mycelia were incubated at 28°C in 250 mL conical beakers containing 100 mL of CYM medium in static culture that was shaken three times a day, for 30 s. Mycelial fragments were collected by centrifugation at 3000 g for 10 min, washed twice with 10.93% mannitol and then resuspended in 1 mL 2% lysing enzymes (Guang Dong Institute of Microbiology, China) containing 10.93% mannitol. After incubation for 1.5 h, the mycelia pellets were washed twice with electroporation buffer (10.93% mannitol, 10 mM sodium phosphate [pH 7]) to remove the enzymes, pelleted by centrifugation at 3000 g for 10 min and resuspended in 1 mL of electroporation buffer per 0.3 g of wet weight of mycelia.

Next, ever 200 µL of mycelia was mixed with 10 µL of plasmid DNA (0.5 µg/µL PEG-purified plasmid pGL-GPE, which contains the *EGFP* and *hph* genes [31], in sterilized water). The mycelia fragments were incubated on ice for 10 min before electroporation. Exponential decay high-voltage electric pulses were delivered by a BTX ECM 630 using 2 mm cuvettes (BTX, San Diego, CA). The electroporation settings used for transformation were 10 kV/cm field strength, 25 μF capacitance, and 400 Ω resistance.

After pulse delivery, the mycelia fragments were incubated on ice for 10 min and mixed with 5 mL of CYM selective medium (37°C) containing 10.93% mannitol, 1% LMP (low melting point) agarose, and 100 µg/mL hygromycin B. The mixture was poured onto a CYM plate containing 10.93% mannitol, 1% agarose, and 100 µg/mL hygromycin B. The plate was incubated at 28°C for 10 d. To estimate viability, small samples of mycelia fragments were cultured on hygromycin B-free CYM plates before and after electroporation.

RNAi vectors were also transformed into *G. lucidum* using these electroporation conditions.

### Detection and stability of the introduced sequence in transformants

The transformants were first selected on a CYM plate containing 100 µg/mL hygromycin B and then subcultured on media without selection for 6 generations (approximately 3 months), or the RNAi transformants were cultured on CYM with 600 µg/mL 5-FOA (SIGMA). Genomic DNA isolated from putative hygromycin B-resistant or 5-FOA-selected transformants was analyzed by PCR. The *hph* gene was amplified using the primers HPH-4 and HPH-5, which were previously used for plasmid construction (table 1).

### Phenotypic analysis of transformants and laccase enzyme assay

Colonies on CYM selective medium were transferred to nonselective medium and incubated for 10 days before they were analyzed by fluorescence microscopy. The green fluorescence emission from EGFP was detected using a Nikon Eclipse Ti–S microscope. Images were recorded and processed using the NIS-Elements F package.

The URA3-RNAi phenotype was tested by growing transformants on MMM with and without uracil (100 mg/L) and also by growing transformants on CYM containing 600 µg/mL 5-FOA for 2 weeks.

Laccase activity on solid medium was measured as described [54] with little modification. The hyphal tip plug was inoculated onto CYM medium supplemented with 10.97% 2,2′-azino-di-3-ethylbenzothiazoline-6-sulfonate (ABTS, Sigma) for 2 days. The laccase enzyme assay was performed as previously described [47]. One unit of laccase activity was defined as a one-point increase in absorbance at 420 nm at 28°C in 1.0 min.

### Real-time PCR analysis of gene expression

The levels of *URA3* and *lcc1*-specific messenger RNA (mRNA) expressed by the wild-type strain *(wt)* and the isolated RNAi transformants were assessed using quantitative real-time PCR.

Aliquots of 0.2 g of mycelia were collected by filtration from the culture medium and frozen in liquid nitrogen. Total RNA was extracted using an RNA isolation kit (TaKaRa, China), treated with RNase-free DNaseI (TaKaRa) and then reverse-transcribed to cDNA using an oligo (dT)17 primer. Subsequently, *18S* [6] and *URA3* transcript levels were determined by quantitative real-time PCR using SYBR Green I on the Eppendorf Mastercycler ep realplex (Eppendorf, Germany). The primer sequences are presented in Table 1.

PCR reactions were performed using the SYBR Green Real Time PCR Master Mix (TOYOBO, Japan) according to the manufacturer's protocol. After an initial denaturation step at 95°C for 5 min, amplification was performed in three steps: 30 s of denaturation at 95°C, 60 s of annealing at 60°C, and 30 s of extension at 72°C for a total of 40 cycles. Identical PCR conditions were used for all targets. Transcript levels were calculated using the standard curve method and normalized against the *G. lucidum* 18S rRNA (*18S*) gene as an internal control. *Wt* mycelia served as the reference sample against which all other transformants were compared; expression of the reference sample was defined as 1.0 and the expression of the *URA3* or *laccase* genes in all other transformants is reported as the fold increase over the reference sample. Post-qRT-PCR calculations of relative gene expression levels were performed according to the 2^-ΔΔCT^ method described by Livak and Schmittgen [55].

### Bioinformatic Sequence Analysis

The theoretical molecular weights of the proteins were calculated using the on-line ExPASy tool http://expasy.org/tools/pi_tool.html. On-line NCBI Conserved Domains Database http://www.ncbi.nlm.nih.gov/cdd [56], and Pfam http://pfam.sanger.ac.uk/ [57] searches were used to identify potential motifs present in GLDCL-1. Multiple sequence alignments were built using M-COFFEE http://www.tcoffee.org

## Supporting Information

Figure S1 **Protein domains analysis of** ***G. lucidum*** **DCL-1 homologue.** This figure shows the 4 domains that characterize the Dicer-2 proteins that were present in the *G. lucidum* DCL-1 homologue fragment. The domains were identified using the NCBI Conserved Domain Database. The domains in the 1544 amino acid fragment were: DEXDc (DEAD Like), HELIC_c (helicase domain), dsRNA binding, and the 2 RIBOc domains.
(PDF)

Figure S2 **DNA and Amino acid sequence GLDCL-1.** The partial DNA and derived amino acid sequence of the gldcl-1 gene. coding regions and amino acids are given in upper case letters. The DEAD domain is shadowed in blue-green, the helicase domain is shadowed in purple, the dsRNA binding domain is shadowed in green and the RNAse 3 domain is shadowed in red and gray.
(PDF)

Figure S3 **The predicted amino acid sequence of** ***G. lucidum*** **DCL-1 and DCL-1 homologues from other fungi**. In the alignment, blue shading to red shading indicates BAD identity to GOOD identity. Important domains are highlighted in colored boxes. The DEAD domain, the helicase domain, dsRNA binding domain and the two RNAse III domains are highlighted in blue-green, purple, green, red and gray boxes, respectively. blue-green, purple, green, red and gray.
(PDF)
