# Supplementary figures and images for: The Development and Application of a Multiple Gene Co-Silencing System Using Endogenous URA3 as a Reporter Gene in Ganoderma lucidum
Source: PLoS One. 2012 Aug 24;7(8):e43737. doi: 10.1371/journal.pone.0043737 (PMC3427163; doi:10.1371/journal.pone.0043737)

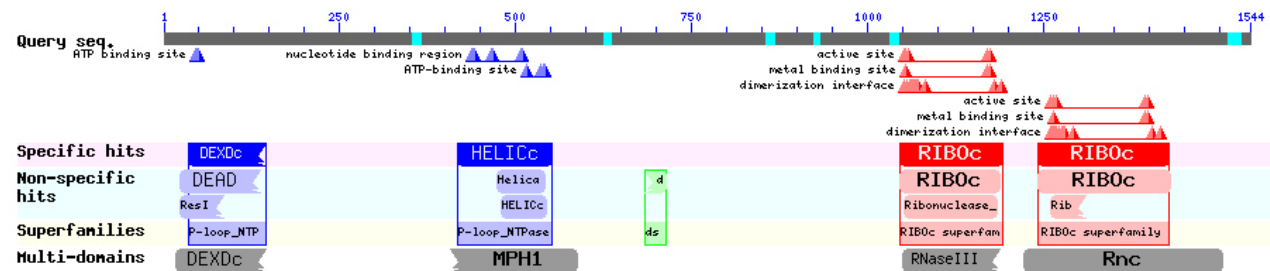

Supplement: Figure S1 — Protein domains analysis of G. lucidum DCL-1 homologue. This figure shows the 4 domains that characterize the Dicer-2 proteins that were present in the G. lucidum DCL-1 homologue fragment. The domains were identified using the NCBI Conserved Domain Database. The domains in the 1544 amino acid fragment were: DEXDc (DEAD Like), HELIC_c (helicase domain), dsRNA binding, and the 2 RIBOc domains. (PDF) [file pone.0043737.s001.pdf]
