# Supplementary material for: The Development and Application of a Multiple Gene Co-Silencing System Using Endogenous URA3 as a Reporter Gene in Ganoderma lucidum
Source: PLoS One. 2012 Aug 24;7(8):e43737. doi: 10.1371/journal.pone.0043737 (PMC3427163; doi:10.1371/journal.pone.0043737)
Supplement: Figure S2 — DNA and Amino acid sequence GLDCL-1. The partial DNA and derived amino acid sequence of the gldcl-1 gene. coding regions and amino acids are given in upper case letters. The DEAD domain is shadowed in blue-green, the helicase domain is shadowed in purple, the dsRNA binding domain is shadowed in green and the RNAse 3 domain is shadowed in red and gray. (PDF) [file pone.0043737.s002.pdf]

1 M P V V K N G D H T G L E E S T S G I V

1 ATGCCTGTTGTCAAGAATGGCGACCACACCGGCCTCGAAGAATCAACGTCCGGCATTGTG

21 H T R A Y Q H E L L E E S L R R N I I I

61 CACACCCGAGCGTACCAGCACGAGCTTCTAGAAGAGAGCTTGCGCCGCAACATCATCATT

41 A L D T G S G K T H I A V L R M K H E T

121 GCCCTCGACACCGGTTCGGGAAAGACACACATCGCGGTCCTCAGAATGAAGCATGAGACT

61 E H E S R K V S W F I A P T V A L V E Q

181 GAACACGAGTCCCGCAAGGTTTCGTGGTTTATAGCTCCTACTGTGGCCCTTGTCGAGCAA

81 Q Y E V I K S T I P V S V G Y V S G S S

241 CAATATGAAGTCATCAAATCTACCATACCTGTTTCGGTGGGATATGTGTCAGGGTCGTCG

101 E P N Q W K D A K L W R R I L Q G H R I

301 GAACCCAACCAATGGAAGGACGCAAAACTTTGGCGGAGGATTCTGCAGGGGCATCGGATC

121 M V T T P Q V L L D A L H H V R I R G Y

361 ATGGTCACAACCCCCCAGGTGTTGCTCGATGCATTACATCACGTTAGGATACGTGGATAT

141 G F Y W F V G S R R S S S R D W Q A P V

421 GGGTTCTATTGGTTTGTTGGTTCTCGACGAAGCTCATCACGCGATTGGCAAGCACCCGTA

161 Q H D H E E P L F V T A A Q R Y R C L N

481 CAACATGATCATGAAGAACCACTATTTGTTACCGCAGCGCAGCGCTATCGATGCCTCAAC

181 F S C T S Y G P R T H S K S Y V W G Q R

541 TTCTCGTGTACGTCCTATGGTCCTCGGACTCACAGCAAGTCCTACGTATGGGGCCAACGT

201 R D C L Q V R A F C S G T T L G F T T N

601 CGAGATTGCCTTCAGGTGCGTGCATTTTGTAGTGGCACAACCCTGGGATTCACCACGAAC

221 L V R C V R D L E K N L D S T I R S S R

661 CTCGTTCGCTGTGTTAGAGATCTGGAGAAGAATCTGGACAGCACCATCCGCTCTTCGCGT

241 N N R D E L A Q H V H R P E F K H I I Y

721 AACAACCGGGACGAACTGGCCCAACACGTTCACCGACCGGAGTTCAAGCACATTATCTAC

261 A S P V H T W D G I P S V N Y K A L S N

781 GCTTCCCCTGTTCATACCTGGGACGGCATCCCTTCTGTCAATTACAAGGCGCTCTCAAAC

281 V M L T L D I E K D P Y V I A L R E R L

841 GTGATGTTGACTTTAGACATCGAGAAGGACCCTTACGTCATTGCCCTTCGCGAACGTCTC

301 A K L Q P G D D R R R V D Q Q L S K A I

901 GCCAAACTCCAACCCGGCGACGACCGCCGCCGCGTGGACCAACAACTCTCGAAGGCCATC

321 D K E D T F T H K G L R D F A R T A A D

961 GACAAGGAGGATACGTTTACACACAAAGGACTACGGGACTTCGCGCGCACTGCCGCAGAT

341 V C I E L G T W A A D W Y V A K V V E H

1021 GTCTGCATCGAGTTAGGGACGTGGGCCGCCGACTGGTACGTTGCGAAGGTCGTCGAGCAC

361 A K K A A N P Y N N L M S A W Q E K E K

1081 GCGAAGAAGGCTGCGAATCCGTACAACAACCTCATGTCAGCGTGGCAGGAGAAGGAGAAA

381 R Y L L S I L E S V D I M E P S N D T G

1141 CGGTACCTTCTGAGCATCCTTGAGTCAGTCGACATCATGGAGCCGTCGAACGATACTGGA

401 E I L A N I T P K V R A L I D N L I L E

1201 GAGATCCTCGCCAACATTACCCCGAAGGTCCGCGCCCTCATCGACAATTTGATCCTCGAG

421 E A I F R S M D E D Y S G L I F A T R R

1261 GAGGCCATCTTCCGATCGATGGACGAAGATTACAGTGGCCTCATCTTCGCGACTCGCCGC

441 D T V L V L A E L L R R I P E T S Q L F

1321 GACACCGTCCTCGTTCTTGCGGAGCTCCTTCGGCGTATCCCCGAAACCTCCCAGCTGTTC

461 R A G C L L G S S S S F K R H S F L D I

1381 CGCGCTGGATGTCTACTGGGTTCGTCTTCGAGCTTTAAGCGGCATTCGTTCTTGGATATT

481 T R S M L E D S Q S D T L R D F K I G D

1441 ACGCGGAGCATGTTGGAGGACAGCCAGAGCGACACCCTGCGGGACTTCAAGATCGGCGAC

501 K N L I V S T S V A E E G I D I Q A C G

1501 AAGAACCTGATCGTCTCTACGTCTGTCGCGGAAGAAGGAATCGACATTCAAGCATGTGGA

521 S V V R F D I P P N V V S W A Q S R G R

1561 AGTGTTGTCCGGTTCGATATTCCTCCCAACGTCGTCTCTTGGGCGCAGAGCCGCGGCCGT

541 A R R K R S S F I I M F D E A A P Q E V

1621 GCGCGCCGGAAAAGGAGTAGTTTCATCATCATGTTCGACGAAGCCGCGCCGCAGGAGGTA

561 V R K W E E T E R Q M M A A Y N D P K R

1681 GTACGGAAGTGGGAGGAGACAGAGCGTCAGATGATGGCGGCTTACAACGATCCGAAACGG

581 D A A A Y I E E D D F N D M D G Y V E V

1741 GATGCCGCTGCTTACATCGAGGAAGATGACTTCAACGATATGGACGGTTATGTCGAGGTC

601 E I A S T G L V S F S S S S K M P A H V

1801 GAAATTGCTTCCACTGGGTTAGTTTCCTTTTCTTCCAGCTCAAAGATGCCAGCTCACGTA

621 P F S G P S R F I R R S R I S I T F A P

1861 CCTTTTTCAGGGCCCTCCCGCTTCATTCGGCGATCCCGCATCTCAATCACTTTTGCGCCC

641 G T R D L G E Q Q L F S H E H F H P S T

1921 GGTACTCGGGACCTTGGGGAGCAACAGTTATTCTCCCACGAGCACTTCCACCCCAGTACG

661 S Y V V M W I S Q R R V S S L R K F K T

1981 TCGTATGTTGTTATGTGGATATCCCAACGTCGCGTATCTAGCCTGCGGAAGTTCAAGACT

681 E C I H R T K R R A Q Q H V A Y K A Y V

2041 GAATGCATCCATCGTACGAAACGCCGAGCGCAGCAGCATGTAGCGTACAAGGCGTACGTG

701 A L Y Q A G L L N N H L L P H T S G V E

2101 GCGCTGTACCAGGCCGGTCTTCTGAATAACCACTTGCTCCCGCATACGAGCGGCGTCGAG

721 P D K E E E V Q A M L A E V A K R E S T

2161 CCTGACAAGGAGGAAGAAGTGCAGGCGATGCTGGCTGAGGTGGCCAAACGCGAAAGTACC

741 A Q V S V Q I D P W V G V A E T S T W F

2221 GCCCAAGTATCCGTCCAAATCGACCCATGGGTTGGTGTCGCGGAGACCTCAACTTGGTTT

761 A H D L V V E G L P S L R M F T R R P L

2281 GCGCACGATCTCGTAGTCGAAGGCTTACCCTCCTTGCGCATGTTCACCCGCCGCCCACTA

781 P T F T Q E D F P T L Y V P G R K E L S

2341 CCGACGTTCACCCAGGAAGATTTCCCGACATTATACGTTCCTGGCCGCAAGGAGCTGTCA

801 M T L G A A A S S P A S V E D I Q L A R

2401 ATGACCCTCGGTGCTGCGGCTAGCTCTCCTGCGTCCGTGGAGGACATCCAGCTTGCAAGA

821 R W T Y R L F W R M Y G A R M E R G N E

2461 CGTTGGACGTACAGGCTTTTCTGGAGGATGTACGGAGCCCGGATGGAGCGCGGCAACGAA

841 D F S Y L F L P T G P W P N A Q E W D E

2521 GACTTTTCGTACCTCTTCTTGCCTACGGGACCTTGGCCGAACGCGCAGGAGTGGGACGAG

861 R R R W M E E R L M S G L A D H G E T P

2581 CGACGGAGATGGATGGAAGAGCGACTCATGTCAGGCCTTGCAGACCACGGGGAGACTCCA

881 F L A N A A V F G A A F S Y P R N L A M

2641 TTTTTGGCGAATGCTGCGGTATTCGGTGCTGCGTTCTCATACCCACGGAATCTCGCAATG

901 V R G T S K Y D K P L R L L Q W R S D P

2701 GTCAGGGGCACGAGCAAATACGACAAGCCTCTGCGGCTGCTGCAGTGGCGTTCCGACCCT

921 L T A E E E E K L R E E Y S A F P D L E

2761 CTCACCGCGGAAGAGGAAGAGAAGCTCCGTGAAGAGTATAGCGCGTTCCCAGATTTGGAA

941 I T Y P L M V V H R L P K R R N F L I P

2821 ATCACGTACCCGCTCATGGTCGTACACCGGCTCCCCAAACGACGGAATTTCCTCATACCC

961 F S T D G T D Q R D P A H S D D G P S I

2881 TTTTCTACTGACGGCACGGACCAGAGGGACCCTGCGCACTCCGACGACGGTCCGTCCATC

981 L L P Q H T L V E L A S R D E V Q Y T L

2941 CTCCTTCCTCAGCATACCCTGGTCGAGCTCGCATCCCGAGACGAGGTTCAGTATACCTTG

1001 V L P S I L R W L A M A S T V H S L R L

3001 GTTCTTCCGTCTATCCTGCGGTGGCTAGCCATGGCATCAACCGTGCATAGCCTTCGTCTG

1021 G L F K Q S P V S R V A L P L L A T A I

3061 GGCCTCTTTAAGCAATCTCCGGTTTCACGCGTCGCGCTCCCGCTCCTCGCTACTGCCATC

1041 T A P T A Q E R F H Y Q R L E T L G D T

3121 ACTGCACCCACCGCTCAAGAGCGTTTTCACTACCAGCGACTCGAGACCCTTGGCGACACG

1061 V L K F I T S N Q L Y A N F P L W H E G

3181 GTGTTGAAATTCATTACAAGTAATCAACTCTACGCGAACTTCCCTCTCTGGCACGAAGGG

1081 Y L S R K K D H A V A N V N L A K W A V

3241 TACCTCAGTCGGAAAAAGGACCACGCCGTCGCCAACGTCAACCTGGCTAAATGGGCCGTG

1101 Q K G L Y R W I I R D R F V P R K W K P

3301 CAGAAAGGGCTGTACAGGTGGATCATCCGTGATCGATTCGTCCCAAGGAAGTGGAAGCCC

1121 H Y S T C N I D E I V D D S T E S T P P

3361 CATTACTCTACATGCAACATCGACGAGATCGTGGACGACAGCACAGAGAGCACACCTCCA

1141 P N G A V T L I Q S N G K D D E K K Q S

3421 CCGAATGGCGCTGTGACGTTGATTCAAAGCAACGGCAAAGACGATGAAAAGAAACAGAGC

1161 A E E L S T K M L A D V V E S L I G A A

3481 GCTGAAGAGCTCTCGACCAAGATGCTTGCGGACGTCGTGGAGTCCCTCATCGGCGCGGCT

1181 Y E Q G G F D L A I D C A A A F D L G L

3541 TACGAGCAGGGCGGATTTGACCTCGCGATCGACTGTGCTGCGGCATTCGATCTCGGTCTC

1201 T W K K L P T Y V T A M H E V D D I D D

3601 ACCTGGAAGAAACTCCCGACATATGTTACAGCCATGCACGAGGTTGACGACATCGACGAT

1221 I P E Q L E L V E Q M I D Y R F S R R T

3661 ATTCCCGAACAGCTCGAACTGGTCGAGCAGATGATCGACTATCGCTTCTCCCGTCGTACT

1241 F L V Q A L T H A S Y Q G E S A S M S L

3721 TTCCTCGTGCAGGCCCTCACCCACGCGAGCTACCAAGGCGAGTCCGCCTCGATGTCGCTC

1261 E R L E F L G D C A L D M V V V D Y L Y

3781 GAGCGGCTTGAGTTCCTCGGCGACTGCGCCCTCGACATGGTCGTCGTCGACTACCTCTAC

1281 H A E G K D Y S P G Y M H I K K M A V V

3841 CACGCAGAAGGCAAGGACTACAGTCCGGGCTACATGCACATCAAGAAAATGGCGGTCGTG

1301 N S H I L A Y I C L K T S V T V Q S T M

3901 AACTCGCACATCCTCGCGTACATCTGCCTCAAGACGTCCGTCACCGTCCAGTCGACGATG

1321 P T W S L A H G L E S V D D S Q Q I H L
3961 CCGACGTGGTCGCTCGCGCACGGGCTGGAGAGCGTCGACGACTCGCAGCAGATCCACCTC
1341 W Q C L L H S S H R V L E D Q N L T F L
4021 TGGCAGTGCCTCCTCCACTCGAGCCACCGCGTGCTCGAGGACCAGAACCTCACCTTCCTC
1361 R F Q K H G A E I A N A L E H H S V Y P
4081 CGCTTCCAGAAGCACGGCGCGGAGATCGCGAACGCGCTCGAGCACCACAGCGTGTACCCG
1381 W A G L T S L Q A P K F I S D M V E S I
4141 TGGGCGGGGCTGACGAGCCTCCAAGCGCCCAAGTTCATCAGCGACATGGTCGAGAGCATC
1401 L G A V Y V D S R G D L G K V R Q V M E
4201 CTCGGGGCGGTGTACGTCGACTCGCGCGGGGACTTGGGCAAGGTGCGGCAGGTGATGGAG
1421 R L G L M R V L E R V V H D D V D A L H
4261 CGGCTTGGGCTGATGCGAGTGCTGGAGCGCGTCGTGCACGACGACGTGGACGCGCTCCAT
1441 P V S R L E I W A A R H K P K K G V K Y
4321 CCCGTGTCGCGCCTAGAGATTTGGGCCGCGAGACATAAACCGAAGAAGGGGGTCAAGTAC
1461 E I E K E K G K V H C K V L I D E K E L
4381 GAGATCGAGAAGGAGAAGGGGAAAGTGCACTGCAAGGTGCTGATCGACGAGAAGGAGCTG
1481 L T V T R T Y R S K V S E D E V R F T A
4441 CTGACGGTCACGAGGACTTACCGGAGCAAGGTCAGCGAGGACGAGGTGCGGTTTACCGCA
1501 A E E A N K M V L A G D I D F D K I E D
4501 GCGGAGGAGGCGAACAAGATGGTTCTGGCAGGGGACATAGACTTCGACAAGATCGAAGAC
1521 D Q S D D E E E Q V E A I L A N R E E D
4561 GACCAATCAGATGACGAAGAGGAACAGGTCGAAGCGATCTTGGCCAACCGCGAGGAGGAC
1541 E V R N -
4621 GAAGTGAGGAACTAG
