# Supplementary material for: The Development and Application of a Multiple Gene Co-Silencing System Using Endogenous URA3 as a Reporter Gene in Ganoderma lucidum
Source: PLoS One. 2012 Aug 24;7(8):e43737. doi: 10.1371/journal.pone.0043737 (PMC3427163; doi:10.1371/journal.pone.0043737)
Supplement: Figure S3 — The predicted amino acid sequence of G. lucidum DCL-1 and DCL-1 homologues from other fungi. In the alignment, blue shading to red shading indicates BAD identity to GOOD identity. Important domains are highlighted in colored boxes. The DEAD domain, the helicase domain, dsRNA binding domain and the two RNAse III domains are highlighted in blue-green, purple, green, red and gray boxes, respectively. blue-green, purple, green, red and gray. (PDF) [file pone.0043737.s003.pdf]

```
M-COFFEE SCORE=86
        BAD AVG GOOD
G.l                                :  83
Paracoccidioides brasiliensis      :  89
Trichophyton rubrum CBS 118892     :  91
Aspers terreus NIH2624             :  85
Arthroderma otae CBS 113480        :  84
Aspergillus niger CBS 513.88       :  87
cons                               :  86

G.l                  1 ----------------------------------------------------------MPVVKNGDHTGL--EESTSGIVHTRA  24
Paracoccidioide      1 ----------------------------------------------------MSQAAKYPVIDSALKNGIGLDSYAASVFTPRA  32
Trichophyton         1 MPIKTHQEHLSTTTAATKRRRQQRCMATAKPELPGQMLALEEHKQGRKRPRSSSGSSDMRQNEAAQKAGLDSLEDAPDMMRPRG  84
Aspers               1 ------------------------------------------------------------------MASLSDQDLEMGLFCPRN  18
Arthroderma          1 MAA-------------------------VELDLHSQKFLLEEHGQKRKRPSSSLGSSEMPQDESAGGASLNGQEGASAVVKSRA  59
Aspergillus          1 ---------------------------------------------------------------MTVAATVLPAGEDAPAYRPRS  21
cons                 1                                                                      :           .*   84

G.l                 25 YQHELLEESLRRNIIIALDTGSGKTHIAVLRMKHETEH-ESRKVSWFIAPTVALVEQQYEVIKSTIP-VSVGYVSGSSEPNQWK 106
Paracoccidioide     33 YQLEMLEASLKENIIIAMDTGSGKTQIAILRIRHELETCTEDKLVWFLTPTVALADQQHKNISQQLSVYQTRLLLGTDKVHYWS 116
Trichophyton        85 YQREMLEASIKQNIIVAMDTGSGKTQIAILRIRHELERCSNDKLVWFLAPKVPLAEQQYRAISEQLPAYQTKILTGADNLERWS 168
Aspers              19 YQTEMFEASLQENIIVAMDTGSGKTHVALLRIMNELETRRPQKLIWFLAPTVALCFQQHDVITKNLPAVKSRTLTGQDKVELWT 102
Arthroderma         60 YQVEMLEESLKQNIIVAMDTGSGKTQIAILRIRHELERCPIHKIVWFLAPKVPLAEQQYLAISKQLPAYQTRILTGADNLERWS 143
Aspergillus         22 YQVEMFEASLKENIIVTMGTGSGKTHIALLRIIKELESN-PHKLIWFLTPTVALCLQQFKFLSDNIPAVRARTLTSLDKVELWT 104
cons                85 ** *::* *::.***:::.******::*:**: :* *     *: **::*.*.*  **.  :.. :.      : . .: . *. 168
```

```
G.l              107 DAKLWRRILQGHRIMVTTPQVLLDALHH--VRIRGYGFYWFVGSRRSSSRDWQAPVQHDHEEPLFVTAAQRYRCLNFSCTSYGP 188
Paracoccidioide  117 TKKIWDDVLLNIKIVVSTPQILLDALSHGFITMSRISLLVFDEAHHCDGFSPANTIMQRF---------------------YHE 179
Trichophyton     169 TQQIWDAFLLNTRIVVSTPQILLDVLSNGFITLRRIALLVFDEAHHCVKASPENKIMKNF---------------------YHA 231
Aspers           103 EQAIWDAILKDMQVVVSTHAVLADAMSNGFVRVSQLGLLIFDEAHHCMRRHPANRIMQDF---------------------YHP 165
Arthroderma      144 TQKIWDAFLLNTRIVVSTPQVLLDALSNGFITLRRIALLVFDEAHHCVKSAPENRIMQNF---------------------YHA 206
Aspergillus      105 EQPVWDAILKEMQVVVSTHAVLADAMSHGFVKITQLGLMIFDEAHHCMRRHPANKIMQDF---------------------YHP 167
cons             169    :*  .*   :::*:*  :* *.: :  : :   .:  *  :::.        : : .                     *   252

G.l              189 RTHSKSYVWGQRRDCLQVRAFCSGTTLGFTTNLVRCVRDLEKNLDSTIRSSRNNRDELAQHVHRPEFKHIIYASPVHTWDGIPS 272
Paracoccidioide  180 QLQ----RTGTKTDLPHILGLTASPVTRADP---ESLKRVEKNLNARCETPRIHREELMRYVHRPELYTVKFRT----DSLIHS 252
Trichophyton     232 RID----QLSETNDLPSILGLTASPTSRLIE---DSLRQLEQNLDAFCKTPAIHREEMMQYVHIPELLKVSYQK----DSIIPH 304
Aspers           166 TLV----KQG-PDAVPGILGLTASPVVRTNS---QELSMIESNMNAICKTPRAHRQELLTHTHRPHLQQIWYTPVNIDDPTSGT 241
Arthroderma      207 QVH----QLRKTNDLPYILGLTASPTSSSAE---DALRQLELNLHACCKTPNIHREEMMQFVHIPELRKVSYQK----DLMLLN 279
Aspergillus      168 ALE----RHG-AEAVPKILGLTASPVVRSNR---QELLKIESNLDAVCKTPRTHRSELMTHTHRPHLQQILFTPVLLDDLQVGS 243
cons             253                  : .: :...          :  :* *:.:  .:.  :*.*:  ..* *.:  : :             336

G.l              273 VNYKALSNVMLTLDIEKDPYVIALRERLAKLQPGDDRRRVDQQLSKAIDKEDTFTHKGLRDFARTAADVCIELGTWAADWYVAK 356
Paracoccidioide  253 DMLQNLSRIVGTVDIEKDPWIKRLRRQE--------DRRSQEKLLYALEKRKTHCLSQLSKLLRQAINVHDDLGPWAADAFISE 328
Trichophyton     305 NMKMKYLHMLDDIDIESDPFFQRYKGKT--------DRKSTERFLCALA-RKTPCLDQLKRCFTKVSHMYGELGHWASSTYISE 379
Aspers           242 RTLRALIHAWETIDLEDDPYVKQLRRST--------F--DGKALQKALLTRKTYCNESLRRFVERSCHIFQELGGWAVDYFIHA 315
Arthroderma      280 NLEVKFFAMLNSIDIQDDPFFQRYKGKH--------DRKSQERYTLALE-RKTPCLIQLKRCFSKIVYMFSELGLWAASTYISE 354
Aspergillus      244 KTLKALVSAWTSLRLEDDPYIKKLRKSP--------L--DGRALQKVLESGKTYCNDQLKRFATRSLHIFEELGEWAADYFIHA 317
cons             337             : ::.**:.   :                .    .:   .*     *         :  :** ** . ::   420

G.l              357 VVEHAKKAANPYNN--LMSAWQEKEKRYLLSILESVDIM--EP-SNDTGEILANITPKVRALIDNLILEEAIFRSMDEDYSGLI 435
Paracoccidioide  329 VIERLETKRTMYQHVELHSSLDREEEAFIFELLSELSISPVKQ-DWDSKSN--IVSTKVNLLVELLESEH------TADFTGII 403
```

```
Trichophyton      380  IYRRTREKRAKLVDQ-NWSEWDRDDSSFMCNALEPVVAIMGER-CWNSTPD--AVSPKVEHLVDLLSSEL------TGASRGII  453
Aspers            316  SIRRLREK-I--DDSALMLDWDNEEKEYLASFLSNIATIQSDPPRRPEDFI--P-SPKLEALISFLSSTD------DSTFSGLI  387
Arthroderma       355  IYRRAHDKKAKLIDQ-NWSEWDKDDASFICNAMLPVIEIMGER-HWDTAPD--SVSQKVDHLIHLLSSEH------TGTSRGII  428
Aspergillus       318  SIEQLKAR-A--GNSADTMGWTDEEKAYLLDIVSKLPIPNIDLTHSDPDRI--PISSKFRSLLEFLDTKG------EPNFSGLI  390
cons              421    .: .      .      .:  ::  . :  :     .          : *.   *:  *              *:*  504

G.l               436  FATRRDTVLVLAELLRRIPETSQLFRAGCLLGSSSSFKRHSFLDITRSMLEDSQSDTLRDFKIGDKNLIVSTSVAEEGIDIQAC  519
Paracoccidioide   404  FAQQRATVTMLSHLISKHPRLKDIIVTGAFLGDACYASRTSTI--TEVHDTRSQKGSIDDLRSGKKNLLIATSVLEEGIDVSAC  485
Trichophyton      454  FVEQRATAVMLSHLIAHYPELAHIKS-DYFLGNSAFSDRKADI--TEISKPGDMKDSIDDLKSGKKNLLVATSVLEEGIDVSAC  534
Aspers            388  FAKQRATVTVLATLLSVHPLTKDRFRCAAFVGWSGGGNRKDLI--GELLSMQMQRDTLSEFRSGQKNLIVATDVLEEGIDISAC  469
Arthroderma       429  FVEQRATAVMLTHLISQHPKLTHIKS-DYFLGNSAFAARKSDI--TELSKPGDMKDSINDLKSGKKNLLVATSVLEEGIDVSAC  509
Aspergillus       391  FAKQRATVSVMEKLLSIHPVTKHRFRCASFVGWSGGGS-KDVL--GELLDARMQRDTLSEFRTGQKNLIIATDVLEEGIDISAC  471
cons              505  *. :* *. ::  *:   *     .      .:* :       :   .       .:: ::: *.***:::*.* *****:.**  588

G.l               520  GSVVRFDIPPNVVSWAQSRGRARRKRSSFIIMFDEAAP-QEVVRKWEETERQMMAAYNDPKRDAAAYI-EEDDFNDMDGYVEVE  601
Paracoccidioide   486  HLVVCFDAIKNLRSFIQRRGRARKERSKFVM-FLDSDK-ISEEKQWTRLEEVMRSIYEDDMRRLEDVMARENIVEEGDQY--LR  565
Trichophyton      535  DLVVCFDPPKQLRSFVQRRGRARKKSSKFVI-FYAEDD-TATYKDWEAMEDIMKERYLSNKEFIDRL--QEEDDEDEIEYESFR  614
Aspers            470  SVVICYDKPANVKSFVQRRGRARRKESTFAI-LFSTDDELCDLRKWQLLEEAMIEAYQDDERKRCEALALETMAEVVTER--FE  550
Arthroderma       510  DLVVCFDPPKQLRSFVQRRGRARKANSKYVI-FHAEDD-TSTKKDWEAMEDIMKERYSNNKELIDEYLAQESDDEDENDYENLR  591
Aspergillus       472  SVVVCFDKPPNLKSFVQRRGRARHRQSTYAI-MFATDDESSALSKWEDLEQAMIEAYEDDERRLREAWALEAINEEVVER--LE  552
cons              589    *: :*    :: *: * *****:   *.: : :           .*   *  *   *  .  .       *    :      ..  672

G.l               602  IASTG-LVSFSSSSKMPAHVPFSGPSRFIRRSRISITFA-PGTRDLGEQQLFSHEHFHPSTSYVVMWISQRRVSSLRKFKTECI  683
Paracoccidioide   566  IESTGALLTLENARQHLEHFCATLHYA-FTDSRPHFIFDESEGDGTIAAKVVLPNVLDP--------------KFRVIYGSKR  633
Trichophyton      615  IESTGALLTLANARAHLSHFCSTIPCE-FTDTQPDFIISKSGIKDMLTAKVLLPTVLDL--------------QFREFEGIQA  682
Aspers            551  VESTGAVLTADTAVARLHHFCSILPQQPYVDNRPELSFEYDGT-GRRRGTFKLPSCVHP--------------DVRRTRGEKW  618
```

```
Arthroderma       592 IESTGALLTLDNARQHLSHFCSTLPCD-FTDIQPDFIISKAVAGTMFTAKVLLPTALDP---------------QFREFEGIRT 659
Aspergillus       553 VQSTGAVLTADTAVAHLNHFCAVLPRQPYASNEPEFSYEKDDA-DLLRGTVTLPSCVHP---------------GVRRIQGQRW 620
cons              673 : *** :::  .:     *.           .  :              .     ..                .*        756

G.l               684 HRTKRRAQQHVAYKAYVALYQAGLLNNHLLPHTSGVEPDKEEEVQAMLAEVAKRESTAQVSVQIDPWVGVAETSTWFAHDLVVE 767
Paracoccidioide   634 WKTERMAQRDASFQAYLKLYNEGLVNDYLLPVHWRGDEDPK------LEYCEQRPSVVKISRHFDPWSTIA--SRWETTQKFYQ 709
Trichophyton      683 WKREKMAKRDAAFQAYLQLYEVGLVNDHLMPEHCR-TTDEE------TAHVEKRTSMATCSEVFNPWKMVA--YRWHSTDTFYQ 757
Aspers            619 WTTERAATKEAAFQTCKRLYEFGLLNDHLLPLTRK--PELR------LTDFGGLPSIIEVAEQYDPWTDWAY--SWSSPD-IHQ 691
Arthroderma       660 WKKEKMAKRDAAFQAYLQLYEVSLVNEHLMPEHCH-TTDEE------TAHIEKHPSTATCSEAFSPWETIA--DQWQATDTFYQ 734
Aspergillus       621 WQTERAARKEAAFQAYKRLYEFGLLSDHLLPFKRN--LELK------ETDLTNLPALVEVSEQYDPWVDWAC--SWSSPD-VHQ 693
cons              757    :: * :..::::   **: .*:.::*:*       : .              :    :   .**   *    * : : . : 840

G.l               768 G----------LPSLRM--FTRRPLPTFTQEDFPTLYVPGRKELSMTLGAAASSPASVEDIQLARRWTYRLFWRMYGARMERGN 839
Paracoccidioide   710 TLIEISSDSIPFPQMRL--VLPVPLPC---DISFNIFWNENNTFEVHLK-QESSSITASLIKHAAQATHAIFSSMFSHKMPSDR 787
Trichophyton      758 SSIVIASGTEELPRMLL--ILPIPLPC---DFTVKLFWNEKSTLLASVS-PQELTVSDEDIVSAPLATHILLSSVFPSRMNGTS 835
Aspers            692 SRIRVQLNGNPEYQLSMSLMGPTVLPA---LDAMTLFWDSQNIFTLAFDAAQRVPLVPGDVIEHMRAITALYLQAPSSRSIREE 772
Arthroderma       735 SSIVISSGTEELPKMLL--ILPAPLPC---SFTFKLFWNETSTLSVSVS-PEALTISDKDITSAPLATHILLSSVYSSRMNGDS 812
Aspergillus       694 TRIAIKHNGDS--RMCIRLTSPTSLPP---VEPMTLFWDSETIYTLDFDKPKRMKEIAAESIENMRLATALYLQAASSRQMRPE 772
cons              841               : :       **        .::    .     .                      :       :      924

G.l               840 EDFSYLFLPTGPWPNAQEWDERRRWMEERLMSGLADHGETPFLANAAVFGAAFSYPRNLAMVRGTSKYDKPLRLLQW-----R- 917
Paracoccidioide   788 FDFSCLFLPEVELTE----DEMMKWYSSVQGKIQA----------SDASHYDIESLSKLGLVRRCDRLERPWTAERYRWMKPFS 857
Trichophyton      836 RDFSCIFAPDLAEGH----GGLESWCTKVSESILG----------RNIVSGGLSSIGDFGLARRTDIYARAWVAEKFVWMKRVR 905
Aspers            773 RDYVALFGPDL--PH----TELGAWLLKNGGNDTA----------LDVYSRQVASP-TMGIVRDRTRYDEPLLFKKW-----VV 834
Arthroderma       813 RDFSCVFVPALDKGY----SGLKEWCTKVNKSILG----------KDIENYNLSPV-DFGLARRTDITARPWTVDKFVWMKPVM 881
Aspergillus       773 QDFVTLFGPDL--TD----LELAEWLNKHAGDEPA----------LEVYSRKDFPT-VMGIVRDRSRYNEPMLFKRW-----VV 834
```

```
cons                 925  *:  :* *               *  .   .  .                       :.:.*      ..    ::        1008
G.l                  918 SDP-LTAEEEEKLREEYSAFPDLEITYPLMVVHRLPKRRNFLIPFSTDGTDQRD-----P-AHSDDGPSILLPQHTLVELASRD  994
Paracoccidioide      858 EGARSDDVDGRSGVDE-EE-----I--LHIEGTALPKRTDFLHPVAKSAGPHLH-----S-RT----KKCYPARECSIDKLPLE  923
Trichophyton         906 VED--DSESAEDEEEE-GE-----I--LHVEGEKWPKRADFLHPVTNSTGSKQH-----H-TA----RNCIPARQSCISMLPVG  969
Aspers               835 TED--G---------D-VA-----V--VEMECHSLPKRRNLLQRQTLAQGEIVTTAVDTAPAK----ARIIPATACTIDRLPFT  895
Arthroderma          882 SKN--DGECEEDEEEKVEE-----I--LHIEGEKWPKRTDFLHPVANTDNSKPH-----H-TG----RKCDPVRDSSISMLPVG  946
Aspergillus          835 SGQ--D---------D-TP-----I--VELECDAVPKRRNLLHRQTLAAK-QPDSETPAISSK----IRLILAENCTIDKLPYA  894
cons                1009                .        :    :     *** ::*   :                               :.  .   1092

G.l                  995 EVQYTLVLPSILRWLAMASTVHSLRLGLFKQSPVSRVALPLLATAITAPTAQERFHYQRLETLGDTVLKFITSNQLYANFPLWH 1078
Paracoccidioide      924 YTMFSLLCPSIIHKV--ETFLIAEELNKTILSPVGFTDLNLVITALCAPDAREATNYQRIEFLGDSILKFHTSLQLLAANPIWH 1005
Trichophyton         970 FAKFALFIPSLIHTI--GRYLLAEELSKTILSPIGFQNIQLVLTAITAASAREDINYQRLEFLGDSALKLHASMQLLAEHPLWH 1051
Aspers               896 DTIFGLFISAILDRL--EATLIATRLCETILQDVQFSSTRHVITAISAPTAQSPTDYQRYEFFGDSVLKFTVSCQLYMQHPNWP  977
Arthroderma          947 FSKFALFIPSLIHTV--GRFLLAEELSKTILSPIGFKDIQLVLTAISASSARESTNYQRLEFLGDSALKLHTSMQLLVDHPFWH 1028
Aspergillus          895 ETIFGRFISVILDRL--EATLVATRLCETILRDLEFSSIRHIITAITAPSAQSLTNYQRYEFFGDSVLKFTVSCQLFFQHPNWH  976
cons                1093    :  . . ::  :     : : .*       :       : **: *. *:.  .*** * :**: **: .* **    * *  1176

G.l                 1079 EGYLSRKKDHAVANVNLAKWAVQKGLYRWIIRDRFVPRKWKPHYSTC-NIDEIVDDSTESTPPPNGAVTLIQSNGKDDEKKQSA 1161
Paracoccidioide     1006 EGLLSNAKDEVVSNKRLSYAAIETGLDKFIFMDIFTGVKWRPRYNKA-H-----------VE----------EQ------DIPR 1061
Trichophyton        1052 EGILSKKKDSIVSNSRLWKAAVDTGLDQFILTTCFTGAKWRPLYNST-Y-----------TN----------NAG--KIGEKST 1111
Aspers               978 EGYLSEGRDEIVQNNRLARAALDVGLDAFILTRRFTPRKWTAPLISEKA-----------VE----------V-------A-GK 1032
Arthroderma         1029 EGLLSHKKGFIISNSRLWKATIETGLDRFILTACFTGAKWRPLYNST-Y-----------TA----------NAG--RVGKNST 1088
Aspergillus          977 EGYLSEGRDEIVQNSRLARAALDAGLDAFIMNKMFTPRKWSAPLISEKI-----------SL----------T-------P-KQ 1031
cons                1177 ** **. :.  : * .*   ::: **  :*:   *.  ** .   .                                      1260
```

```
G.l               1162 EELSTKMLADVVESLIGAAYEQGGFDLAIDCAAAFDLGLTWKKLPTYVTAMHE--VDDIDDIPEQLE-L-VEQMIDYRFSRRTF 1241
Paracoccidioide   1062 REMSTKTLADVIEALLGAATIDGGRTKVEKCLKIFLPEIRWMPFADRFDALYNSVPEVYENLPSSWF-RDIESLVGYIFNKKVF 1144
Trichophyton      1112 RELSTKTLADVVESLIGAAIIDGGENKVLHCLELFLPEINWLPSGERIEILYDSALDFNDDCPRDIL-SKVEPLIEYSFTKKAL 1194
Aspers            1033 RPLSSKVLADVVESLIGAAYMDGGQAKAHTCIRRLLPEIEICPIT---------PPPVHESAPHVMN-DSLKQHLGYTFVNEAL 1106
Arthroderma       1089 REISTKTLADVVEALIGAAIVDGGEDKALHCLQVFLPEINWLSYEKRIEMLYDASPEFHDNAPHDIL-SKIEPLIDYSFTKKAL 1171
Aspergillus       1032 RTMSTKVLADVVEALIGASYIDGGFAAAHACIHRFLPEVNLENIDRTT-----APMPKDGVTNHTLNDDHLMAHIGYTFTNKSL 1110
cons              1261 . :*:* ****:*:*:**:  :**   .  *   :   :                               :   : * * .. : 1344

G.l               1242 LVQALTHASYQGESASMSLERLEFLGDCALDMVVVDYLYHAEGKDYSPGYMHIKKMAVVNSHILAYICLKTSVTVQSTMPTWSL 1325
Paracoccidioide   1145 LAQAFTHPSNPG--STMSYQRLEFLGDSILDHIIVDHIFNSP-RNFQHVDMHLMRASLANADFLAFLCIGMTTEEERGEIVEAS 1225
Trichophyton      1195 LAASLSHPSNPI--SGMTYQRLEFLGDSILDNIVTRALFRCK-KEIPHQDMHLIRTALVNADFLAFLCMNTSTDETREDVCVSP 1275
Aspers            1107 LVEALTHPSCRSDASTQSYQRLEFLGDAVLDMVVVHAMAHHA-VECPQGEMTMIKHALTNANLLAFFCMEFVVAQEHTDVDAVP 1189
Arthroderma       1172 LATSLSHPSNPI--SGMTYQRLEFLGDSILDSIVTRALFRSN-KEIPHQDMHLMRTALVNADYLAFLCMNTSTDEQREDVSASS 1252
Aspergillus       1111 LVESLTHPSCQFDTTTQSYQRLEFLGDAVLDMAIMSTLLSHP-REIPQGLMTKIKHAVVNANLLAFFCMEFALTEKRTNVQVTP 1193
cons              1345 *. :::*.*     :  : :*******. **  :   :      :     *   : ::.*:. **::*:                1428

G.l               1326 AHGL-ESVDDSQQIHLWQCLLHSSH-RVLEDQNLTFLRFQKHGAEIANALEHHSVYPWAGLTSLQAPKFISDMVESILGAVYVD 1407
Paracoccidioide   1226 RNGT-FTRMTTRKISLWQYMRHGTSWELTVSQQQTVNQYQALKADITKALYESDTYPWALLSRLRAQKFFSDLIESVLGAIFID 1308
Trichophyton      1276 TGEV-EVTSSTRQVSLWQYMSHSST-DIVETQQAMARTFEELRGQIENALRSGARYPWTLLSTLNAPKFFSDIIESILGAIFID 1357
Aspers            1190 AAGGFALRSEQKPIELWRFMRSEAL-DLNNARETVLHRHSQLRAEIVHALHHGAQYPWQALSQLNADKFFSDIVESILGAIFVD 1272
Arthroderma       1253 IGKV-EITSSTRRVSLWQYMSHSAALDIANAQLATVRKFEELREKIDEALRSGTRYPWTLLLTLNAPKFFSDIIESILGAIFID 1335
Aspergillus       1194 TG-TVTLNPSTEHIELWRFMRYQGA-HLQTARDLALSRHSSLRGSIIHGLKHSPSYPWKSLSQLNADKFFSDIIESILGAIFID 1275
cons              1429            . : **: :       :   :      ..    .* ..*     ***  *  *.* **:**::**:***:::* 1512

G.l               1408 SRGDLGKVRQVMERLGLMRVLERVVHDD-VDALHPVSRLEIWAARHKPKKGVKYEIEKE------K-GKVHCKVLIDEKELL-- 1481
Paracoccidioide   1309 SHGSLDACAAFLERIGLMNFLRRILQDE-IDIMHPKQRLGEMVGQMQVRYETVHVVEEEGGD---ALGHWTCKVFVDDECVF-- 1386
```

```
Trichophyton       1358 SRGSILACQKFLTRIGLMDYLQRVLEDDGIDFMHPKQRLGVVAQSLSVQYVVSEVHVKPG-----V-TQWKCQVLVGDEEIS-- 1433
Aspers             1273 SRGDLDVCAMFVERIGLLPYLRRILADR-VDVMHPRHT-AQRLSKGEALFTAKRVVDGSG------NASYRCVVKRNKEE-I-V 1346
Arthroderma        1336 SRGSVEACQTFLSRIGLMGYLNRVLDDGNIDFMHPKQRLGQIVQSLTVKYIVSDVH-KHG-----V-MRWNCQILVGDQEIC-- 1410
Aspergillus        1276 SHGNLAECEKFLERLGLLRYLRRILKDE-VDVMHPRNI-AQQMAKGEIRFEVLRVPNEGGGGGEDDGATYRCTVKMAGVDGVAV 1357
cons               1513 *:*.:     .: *:**:  *.*:: *  :* :**                .                   * :          1596

G.l                1482 TVTRTYRSKVSEDEVRFTAAEEANKMVLAGDID--FDKIEDDQSDDEEEQVEAILANREEDEVRN 1544
Paracoccidioide    1387 EE----HDGVSLVEVETKAAEAALSVLQNR--------------------------------GAG 1415
Trichophyton       1434 RV----DDGVSQNQARAKAADLALEVLRTE----------------------------------N 1460
Aspers             1347 VV----EGCLSSEEAEVKAANATIGILRANAVN--L----------------------------V 1377
Arthroderma        1411 QI----NDGVSRIHAETKAAEIALEILQTR----------------------------------N 1437
Aspergillus        1358 VV----EGCLTSEEAEITAAERAVEILVEQINASRFL-----------GWVSSSPSNTSSRRSYQ 1407
cons               1597        . ::  ... .**: :  ::                                      1661
```
